# Prioritizing chronic pain self-management amid coexisting chronic illnesses: An exploratory qualitative study

**DOI:** 10.1016/j.ijnsa.2024.100175

**Published:** 2024-01-06

**Authors:** Charlotte Moore-Bouchard, Marie-Eve Martel, Elise Develay, José Côté, Madeleine Durand, M Gabrielle Pagé

**Affiliations:** a Research Centre of the Centre Hospitalier de l'Université de Montréal, Montréal, Canada; b Department of Psychology, Université de Montréal, Montréal, Canada; c Faculty of Nursing, Université de Montréal, Montréal, Canada; d Department of Medicine, Université de Montréal, Montréal, Canada; e Department of Anesthesiology and Pain Medicine, Université de Montréal, Montréal, Canada

**Keywords:** Chronic disease, Chronic pain, Chronic illness, Health psychology, Prioritization, Qualitative, Self-management

## Abstract

**Background:**

In Canada, one out of five people lives with chronic pain, a condition frequently co-occurring with other chronic illnesses. As with most chronic illnesses, successful engagement in symptom management is key. In the context of multiple illnesses, self-management involves daily prioritization of symptoms and conditions and decision-making, which can be challenging. Self-management of chronic illnesses can require more complex competence and tasks to address the different implications of each condition.

**Objective:**

Our research objective was to explore types and processes of self-management symptom prioritization among adults living with chronic pain and other chronic illnesses.

**Design:**

This research was carried out as part of a larger study that adopted an explanatory sequential mixed-methods design. This study focused more specifically on the qualitative part of the study.

**Setting(s):**

Participants recruited for the qualitative component took part in a semi-structured individual interview online or in-person at the center hospitalier de l'Université de Montréal.

**Participants:**

In total, 25 participants were interviewed, including 18 women and 7 men.

**Methods:**

To participate in the qualitative part of the study, participants were selected from the larger study and were eligible if they were 18 years old or older and experiencing pain for more than 3 months and had at least one other chronic illness for which they were receiving treatment or engaged in symptom management. Semi-structured interviews were conducted in-person or virtually and were transcribed verbatim. Reflexive thematic analysis was used to explore patients’ narratives, and an open and iterative approach was adopted to code interviews and generate themes.

**Findings:**

The first theme, focus on symptom prioritization, showed different prioritization processes, including prioritizing a dominant illness, prioritizing multiple illnesses to avoid undesirable consequences, and finally absence of or automatic processes of prioritization. In the second theme, we identified several characteristics of an illness, in this case chronic pain that made it a self-management priority: uncontrollable and disabling nature, omnipresence, unpredictability, unpleasantness, and invisibility to others. In the last theme, we highlighted that some psychosocial factors influenced levels of engagement in self-management and prioritization processes, including social support and the patient-physician relationship.

**Conclusions:**

Chronic pain was the medical condition most often prioritized by participants in their self-management tasks. Because of its characteristics, it was the medical condition that had the most negative impact on day-to-day functioning.

## What is already known about this topic

• The prioritization of chronic illnesses in the context of self-management often favours a dominant disease, which is determined based on various factors such as its severity, the number of associated symptoms, the intensity of these symptoms, the impact it has on daily life, and the short- and long-term consequences associated to it.
• *Subjective* symptoms associated with a chronic illness, such as emotional distress and pain, pose a greater management challenge for individuals than *objective* signs, such as blood glucose levels or blood pressure.

## What this paper adds

• We revealed multiple profiles of self-management prioritization processes, including prioritizing a dominant illness, prioritizing multiple illness to avoid undesirable consequences, and the absence of or automatic processes of prioritization.
• Several illness characteristics influence self-management priority, including the extent to which it is controllable, predictable, omnipresent, unpleasant and invisible.
• Engagement in self-management prioritization is often automatic, which is important to consider when developing and delivering interventions aimed at increasing engagement in self-management in the context of chronic pain.

## 1 Background

Nearly one out of five people experience chronic pain, a chronic illness characterized by a persistent pain that lasts or recurs for more than 3 months (Treede et al., 2015, 2019; Zimmer et al., 2022; Tracey and Bushnell, 2009). Despite its negative impact on individuals, society, and the economy, chronic pain is often underevaluated and undertreated (Campbell et al., 2019). Chronic pain is multifactorial and influenced by biological, psychological, and social factors (Engel, 1977; Campbell et al., 2019). Its management is therefore multidisciplinary and requires the active participation of the individual (Dorflinger et al., 2013). Self-management is emphasized as a central element to improve quality of life and functioning among individuals living with chronic pain. Self-management can be defined as “the intrinsically controlled ability of an active, responsible, informed and autonomous individual to live with the medical, role and emotional consequences of his chronic illness(s) in partnership with his social network and the healthcare provider(s)” (Van de Velde et al., 2019, p. 10). In the context of chronic pain, it requires constant symptom monitoring leading to cognitive, behavioral and emotional responses that determine the self-regulatory dynamic processes (Barlow, 2001). Indeed, chronic pain is often considered only as a symptom, while it has been recognized as a chronic illness in its own right (Tracey and Bushnell, 2009). This chronic illness comes with significant self-management challenges, particularly because subjective disease-related symptoms, such as pain, are more difficult to manage than concrete symptom monitoring such as blood glucose levels (Ancker et al., 2015). This can be complex for many individuals, however, who also have several other chronic illnesses.

Indeed, more than half of individuals living with chronic pain also simultaneously live with other chronic illnesses (Agborsangaya et al., 2012; Desai et al., 2020; Foley et al., 2021). Despite this preponderance, the healthcare system often treats chronic illnesses in silos. This can lead to conflicting recommendations in terms of self-management. A systematic review of the literature pointed out that fragmented care and the absence of umbrella guidelines for chronic diseases create difficulties for individuals in managing and actively participating in their care (Sinnott et al., 2013). Thus, in the context of frequent co-occurrence of chronic illnesses, prioritizing symptoms or illnesses and self-management tasks can be challenging and require more complex self-management competence and tasks (Bratzke et al., 2015). For example, in some situations, managing one illness (e.g., recommended diet to control glucose levels in diabetes) can lead to the temporary worsening of another illness or signs and symptoms associated with that illness (e.g., increasing abdominal discomfort associated with irritable bowel syndrome) (Bayliss et al., 2003).

From a theoretical perspective, the self-determination theory can help us understand why and how individuals engage in self-management (Ryan and Deci, 2000). This theory states that three psychological needs (autonomy, competence, and relatedness) drive motivation and active engagement in many behaviors, such as health behaviors and coping style. The context of multiple co-occurring illnesses also increases the complexity of self-management tasks, which can be broken down into four phases as stated in the Integration of Chronic Illness Self-Management model: seeking effective strategies, pros and cons of those strategies, creating routines, and negotiating self-management tasks so that they fit into one's daily life (Audulv et al., 2012). Decision-making and priority-setting are thus essential processes in the management of many chronic illnesses. In this context, decision-making can be defined as an individual's ability to make choices in the self-management of their chronic illnesses (Bratzke et al., 2015), while prioritization is defined as an individual's ability to give more importance to one of their illnesses, associated signs or symptoms, or specific self-management tasks (Bratzke et al., 2015). In this narrative review on priority-setting, most studies determined that this process often occurs in a way that prioritizes the dominant disease. A disease is perceived as dominant based on various characteristics, such as the severity of the disease, the number of symptoms associated with it, the intensity of these symptoms, the impact of the disease and associated symptoms on the individual's daily life, and the consequences it causes (Bower et al., 2012; Lindsay, 2009; Schoenberg et al., 2009). Since these characteristics can fluctuate over time, the disease considered dominant by the individual may also change over time (Morris et al., 2011). Other factors can also influence individuals’ prioritization of self-management tasks, such as the extent to which a disease influences general outcomes (for example, quality of life, functional status, social roles, or survival rates) (Bower et al., 2012; Fried et al., 2008; Lindsay, 2009), the extent to which the disease can be controlled, and the extent to which symptom evolution are unpredictable (Lindsay, 2009). Furthermore, diseases that tend to exacerbate or trigger other illnesses when left unmanaged, as well as those negatively affecting the management of other coexisting diseases, are often considered central to the self-management tasks (Fix et al., 2014; Morris et al., 2011; Schoenberg et al., 2011). Lastly, some individuals may prioritize an asymptomatic illness, as it can unexpectedly lead to adverse consequences (Schoenberg et al., al.,2009).

Since self-management of illnesses involves symptom management and monitoring clinical signs, it is important to distinguish the management of symptoms and the management of clinical signs (Bratzke et al., 2015; Coleman et Newton., 2005). Symptoms are considered subjective, as they are perceived by the individual only, while signs are defined as objective, as they can be perceived by outside observers, such as physicians and medical teams (Aronowitz, 2001). Thus, several studies highlight the fact that *subjective* symptoms associated with a chronic illness, such as emotional distress and pain, pose a greater management challenge for individuals than *objective* clinical signs, such as blood glucose levels or blood pressure (Aronowitz, 2001; Bratzke et al., 2015).

Some qualitative studies focusing on priority setting have made a major contribution to our understanding in relation to this essential process in individuals living with multiple chronic illnesses (Bratzke et al., 2015). However, none of these studies has examined this self-management process in the context of chronic pain. Considering the high prevalence of chronic pain in Canada, its frequent co-occurrence with other chronic diseases, as well as the difficulties associated with pain management, it is relevant to study self-management processes among individuals living with this illness (Agborsangaya et al., 2012; Treede et al., 2015).

The research aim was to explore types and processes of self-management symptom prioritization among adults living with chronic pain and other chronic illnesses.

## 2 Methods

### 2.1 Study design

This research objective was carried out as part of a larger explanatory sequential mixed-methods study. This research focused more specifically on the qualitative part of the study. Overall, participants in the quantitative component were asked to complete online baseline questionnaires, answer an electronic diary twice per week for 6 weeks to document symptom prioritization and management, and, finally, complete a follow-up questionnaire 1 month later. A subset of participants was invited for a semi-structured interview, which was the focus of this paper.

### 2.2 Participants

Eligible participants for the main study had to meet the following inclusion criteria: living with chronic non-cancer pain for more than 3 months, having at least one other chronic physical or mental health illness, being 18 years of age or older, being fluent in spoken and written French or English, and being able to provide informed consent and complete online questionnaires. Given that guidelines for chronic pain management distinguish between noncancer and cancer-related pain (Dowell et al., 2016), we considered for study inclusion only those individuals reporting chronic pain not associated with cancer. These individuals could have concurrent cancer unrelated to their chronic pain. All participants who completed the electronic diary were eligible for the qualitative component, and a maximum variation sampling approach (see below) was used to select them for an individual interview.

### 2.3 Recruitment

Participants were recruited for the main study from ads published via social and mass media and from patient associations (chronic pain, diabetes, multiple sclerosis, and similar.). Interested individuals were invited to contact the researchers to determine eligibility and participate in the study.

Next, participant recruitment for the qualitative component aimed to diversify the chronic pain prioritization profiles obtained using the ecological momentary assessment administered in the quantitative component (see procedure section)—ensuring representation from participants with stable profiles; i.e., those who always prioritized the same symptom(s) in their self-management tasks and those for whom this prioritization fluctuated greatly over the days. Then, to ensure the diversity of experience, participants were recruited to represent different numbers of chronic illnesses they were living with.

### 2.4 Procedure

Participants took part in a semi-structured individual interview online or in person at the Centre hospitalier de l'Université de Montréal (CHUM), depending on their preference and proximity to the research center. Interviews took place from January 2019 to May 2019. A semi-structured interview guide (see Table 1) was elaborated to explore the various factors associated with fluctuations in participants’ symptom prioritization using main themes and subquestions as needed.

**Table 1:** Semi-structured interview guide.

Main question	Additional prompts
1. Can you tell me about your health?	a. What are the main symptoms associated with your chronic conditions? b. To what extent do these symptoms remain stable?
2. How do you perceive your chronic pain?	a. How do you perceive your chronic pain compared to your other chronic conditions? b. What are the most bothersome symptoms? c. What self-management strategies do you use for your pain?
3. How do you manage the symptoms of your chronic conditions on a daily basis?	a. How do your self-management tasks for your symptoms align across different conditions? b. What difficulties do you encounter in self-managing your symptoms? c. What factors facilitate the management of all these symptoms?
4. How do you determine which symptoms to prioritize daily?	a. What helps you decide what to focus on in self-managing your chronic conditions? b. How often do these priorities change?

Initially, participants were asked to talk about their general health and the various symptoms associated with their chronic illnesses, to get a better idea of their different illnesses. They were also asked to describe their chronic pain, including the symptoms associated with it, and their perception of chronic pain in comparison with other chronic illnesses. Day-to-day self-management of pain and other chronic illnesses was then discussed to gain a better understanding of the challenges involved. Questions on how participants prioritized certain symptoms and the reasons behind these choices were asked to gain further insight into symptom prioritization. Finally, to better understand the stability of symptom prioritization over time, participants were asked how often their priorities changed. During the interviews, a logbook was also used by the interviewers to leave notes for the researchers on their impressions of the interviews and to improve the interview guide. Interview duration ranged from 45 to 90 min. Compensation for the qualitative part of the research was CAN$25, plus a CAN$20 reimbursement for transportation and parking expenses, when applicable. All interviews were conducted in the participant's preferred language (French), recorded, and then transcribed verbatim. They were analyzed in their original language, and selected quotes were translated to English using a forward and backward translation process.

### 2.5 Analysis

Thematic reflexive analysis was used to analyze the transcripts and logbook data, and an open and iterative approach was adopted to code interviews and generate themes (Braun et al., 2019). The lead researcher led the analyses, with frequent meetings with two other researchers (GP, ED), who also analyzed some of the interviews. The involvement of multiple researchers in the data analysis was collaborative and reflexive and aimed to develop a richer interpretation of the data, rather than unanimous agreement on its meaning (Braun and Clarke, 2019). Memo writing was used at all stages of analysis to enable the detailed identification and characterization of themes and their interrelationships, as well as to encourage reflexivity. Finally, a search for deviant cases was undertaken to identify cases that differed from the reported conclusions, so that they could be considered in the results. The analyses were conducted using Dedoose, a Cloud-based application that enables the organization and analysis of research data (Dedoose, 2021).

The inductive approach employed for the reflexive thematic analysis enabled us to derive themes directly from the data, rather than extracting them from pre-existing categories or a predetermined conceptual framework. Moreover, the collaboration among multiple researchers, memo writing, and the search for deviant cases encouraged a more open exploration of participants' experiences.

### 2.6 Participants’ description

The majority of the 25 participant interviewees were women, below the age of 60, and with two or more chronic illnesses in addition to chronic pain (see Table 2). Other than chronic pain, other illnesses included: endocrine and metabolic disorders (diabetes, hypothyroidism), heart problems, mood disorders (depression, bipolar disorder), bone and joint diseases and musculoskeletal conditions (fibromyalgia, osteoarthritis, rheumatoid arthritis, degenerative arthritis, Forestier's disease), respiratory problems (asthma, highly irritable bronchi, lung issues), blood disorders, gastrointestinal issues (stomach problems, irritable bowel syndrome, irritable bowel disease), organ malfunctions (liver and kidney problems), neurological disorders (paralyses and paresthesia), hormonal disorders (testosterone deficiency, low testosterone levels), sleep disorders, dermatological conditions, autoimmune diseases (Raynaud's disease, connective tissue disease), hearing problems (tinnitus) and cognitive problems (memory/concentration/orientation/dyslexia issues).

**Table 2:** Participants description.

Description of the 25 participants in the qualitative component.	
Sex of the participants	
Sex	Number of participants
Men	7
Women	18

Age of participants	

Age	Number of participants

40–49 years old	9
50–59 years old	6
60–69 years old	7
70–79 years old	2
No response	1

Number of comorbidities of the participants	

Number of comorbidities	Number of participants

Chronic pain + 1 comorbidity	8
Chronic pain + 2 comorbidities	6
Chronic pain + 3 comorbidities	6
Chronic pain + 4 comorbidities or more	5

Sample size was determined based on information power, a concept that refers to the fact that sample size depends on study objectives, sample specificity, use of theories relevant to the research topic, interview quality, and analytic strategy (Malterud et al., 2016). Our study had relatively focused objectives with prior studies to orient interview questions but a variety of different chronic illnesses in addition to chronic pain. Monitoring of interview depth and quality, as well as redundancy across participants, was also considered in determining when to stop recruitment. Given the mixed study design, recruitment for the qualitative phase was also dependent on the available pool of participants in the broader study.

### 2.7 Ethical considerations

Regarding ethical considerations, this study was approved by the research ethics board of the CHUM, study #18.012. All participants signed a consent form, ensuring their voluntary and informed participation.

To ensure confidentiality, audio recordings of the interviews were uploaded and stored on the secure server of the CHUM, and then deleted once transcription was completed. The verbatim transcripts were also stored on the secure server of the CHUM and anonymized in a way that no participant could be identified.

### 2.8 Findings

**Fig. 1:**
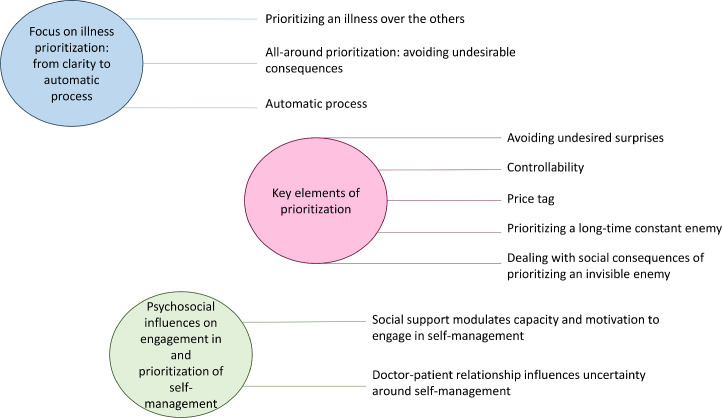
Illustration of study's main themes and subthemes.

This section describes profiles of prioritization styles, how and why prioritization was performed, and the elements that influenced the commitment and execution of prioritization. We identified three main themes that are illustrated in Fig. 1: (1) focus on illness prioritization: from clarity to automatic processes; (2) key elements of prioritization; and (3) psychosocial influences on engagement in and prioritization of self-management.

1. Focus on illness prioritization: from clarity to automatic process

In general, two distinct participant profiles revealed variations in how illnesses were prioritized. For some, they identified specific aspects of their health that they prioritized on a daily basis. This typically led them to focus on one particular chronic illness or symptom as their main focus of their day-to-day self-management tasks.

*Prioritizing an illness over the others.* Several participants made the daily decision to prioritize pain among their other illnesses in their self-management tasks. Indeed, characteristics of pain, described in the next theme, made pain a common focus of attention for self-management. Concretely, many participants first engaged in strategies aimed at reducing pain levels and only then focused on needs of other chronic illnesses. Within- illness prioritization was also described by many, as different types of chronic pain can be present at once. In those cases, the most intense pain was often prioritized. For example, when asked what they prioritize when several pains are present at the same time, one study participant mentioned: “It depends what hurts me the most…” (57-year-old woman living with chronic pain and two other chronic illnesses) It is important to note, however, that this method of prioritizing pain was not adopted by all the participants with multiple pains in this study. Some focused on types of pain they could have a greater impact on. For example, one participant prioritized the least intense pain: “I try to… focus myself, let's say, on the numbing I feel, on the one that hurts the least, let's say… because it's more positive…” (44-year-old woman living with chronic pain and three other chronic illnesses) or gave equal priority to all types of chronic pain when reaching a high enough intensity, thus managing the different pains equally:

“If the pains are really too severe, both of them, well, you know, if it's just numbing, I will prioritize that, but if, let's say, I feel pain in my neck and in my lower back, well then, I would not prioritize any… I would use the TENS [transcutaneous electrical nerve stimulation] machine, you know?” (44-year-old woman living with chronic pain and three other chronic illnesses)

Beyond pain intensity, others discussed how prioritization can also be based on functional outcomes. So, for example, when this individual experienced pain in the arms and legs, he prioritized the legs to be able to walk:

“Because it's a priority, … for me… I have to walk. Of course I also had a past history that… when I was between 11 and 15 years old, my mom got paralyzed… so, she could no longer walk, she could not move at all… so, of course for me, when my legs…. are in pain, well then, I panic a little.” (45-year-old woman living with chronic pain and four other chronic illnesses)

In this example, one type of pain is prioritized due to fear or concern about its impact on functioning. Consequently, this apprehension made lower limb pain a priority since the pain in the arms had a lesser impact on functioning.

Prioritizing pain was not endorsed by all participants, however. Despite the presence of pain, a small number of participants considered one or more other illnesses to be more important and therefore prioritized them in their day-to-day illnesses self-management. For example, one participant in the study reported having pain as well as diabetes among her various chronic illnesses. When asked what she prioritized when pain and diabetes-related symptoms were present simultaneously, she replied:

“I will prioritize my glycemia… I will work on my eating habits… Yes, I will work on my nutrition… to help so that it does not go back up, or you know, that it doesn't go down too much… You know, if it goes down too much, well, introducing more snacks, if it goes up too much, well, I try to go for a walk, I try to… eat better… you know, stuff like that…” (54-year-old woman living with chronic pain and four other chronic illnesses)

The characteristics that influenced the prioritization of these other illnesses are also described in the next theme.

*All-around prioritization: avoiding undesirable consequences.* For other people, several illnesses were simultaneously prioritized since they were considered equally important, and ignoring one to the benefit of another could have important undesirable consequences. For example, a participant with diabetes, asthma, and pain explained that none of these illnesses could be prioritized over another since they could all have undesirable consequences:

“Oh my God! Excuse me, but I cannot prioritize… I don't have a choice, they are all at the front…. If I don't take my Tylenols, you know… it will happen… after that, if I don't take my medications, the diabetes, I will end up in hyper- or hypoglycemia… My asthma, I have to take medications, if I don't have to have another asthma attack, so all three take up an important place daily.” (57-year-old woman living with chronic pain and two other chronic illnesses)

For others, they appeared initially puzzled by questions asking them to explicitly describe how they prioritize the management of their different chronic illnesses.

*Automatic process.* Another interesting finding was that many participants expressed that they did not think about prioritization on a daily basis daily and that this was done automatically. Most of these individuals said that they let their symptoms decide the orientation of their self-management on a day-to-day basis so that their prioritization depended more on the symptoms present at a given moment: “I must admit…, I don't know if I put priorities… I never… I never questioned myself about this really… I take things more as they come.” (74-year-old man living with chronic pain and six other chronic illnesses)

2. Key elements of prioritization

In this section, we explore the different characteristics of illnesses and the related impacts that determine which illnesses tend to be prioritized for self-management.

*Avoiding undesired surprises.* According to many participants, a central element in the decision to prioritize an illness was the degree to which it was unpredictable. Pain is often unpredictable, and this aspect of the illness was mentioned by many as one of the reasons why pain was always prioritized. Unpredictability of pain makes it difficult to plan activities since levels of functioning depend on fluctuating pain intensity:

“It's really minute by minute…, I cannot… plan anything. I cannot control anything about my pain, it comes really… like totally out of left field, and it determines itself how strong, if it will be really painful or not and how long.” (45-year-old woman living with chronic pain and four other chronic illnesses)

This unpredictability led people to avoid or engage in preventive actions to minimize pain levels by prioritizing pain in their day-to-day management tasks, even when pain was not intense at the time of self-management.

*Controllability.* Beyond unpredictability, controllability of an illness was also determinant in prioritization of symptoms and illnesses. A chronic illness that is more difficult to control required extra attention to optimally manage it. Unlike other illnesses, pain was often described as uncontrollable by study participants: “Of course. Yes, of course it's (the pain) the first, because I know that the other ones, I can better control them” (45-year-old woman living with chronic pain and four other chronic illnesses). Indeed, there was not necessarily a protocol to follow to reduce the level of pain, which was more difficult to manage than other illnesses, and thus led to more consequences on the person's daily life. For this reason, pain was often prioritized among other chronic illnesses. Although pain was the illness most often reported as uncontrollable by our study participants, for a small number of participants, other chronic illnesses were considered more difficult to control than pain. For example, for some study participants, diabetes was very difficult to manage or control, as there were many steps to follow precisely for self-management of this illness. In these cases, chronic illnesses considered more difficult to control would tend to be prioritized, rather than pain.

“It's quite a challenge every day to make sure that you control the glycemia, it's the foundation, the secret, to of course avoid in the long-term all of the complications possible that diabetes can bring, and even… it is not 100% guaranteed…” (60-year-old man living with chronic pain and eight other chronic illnesses)

Some participants tended to prioritize illnesses for which there were fewer effective strategies, such as fibromyalgia, and leave out illnesses that were already controlled by well-established strategies in their daily lives, like diabetes. Other participants also appeared to be influenced by the success of their self-management tasks or strategies when prioritizing a chronic illness. For many illnesses, certain self-management tasks were more effective, such as medication, diet, and similar items. Frequently, there was a protocol to follow that worked almost every time for managing these chronic illnesses:

“I would say that it's really the fibromyalgia that is the central part of the problem… in my day-to-day life because, of course, the diabetes, well, I have to pay attention to what I'm eating, so, I try to not put myself in danger, not in hypo- and not in hyperglycemia… It's on me to control it. After that, you know, hypertension is controlled using medications.” (45-year-old woman living with chronic pain and four other chronic illnesses)

*Price tag.* A third factor that seemed to influence the illness prioritized in self-management tasks is the higher price tag of ignoring an illness. In fact, certain chronic illnesses can have serious consequences if poorly managed. For example, uncontrolled or poorly controlled diabetes is likely to have negative direct impacts on a person's health, such as a rise in blood sugar levels that can put the person's health at risk. This is in contrast to pain, which can limit functioning but typically does not have multi-system repercussion when it flares up. To avoid serious impacts associated with poor management of these chronic illnesses, it was important for study participants to prioritize the illness with the most serious consequences if poorly managed.

“It's a challenge every day to make sure I control my glycemia, it's the foundation, the secret, obviously, to avoid long-term complications possible that the diabetes can bring and even, it's never 100% guaranteed… even if we can control it well, with medications, food… exercise, there are still cornerstones of controlling this disease. So, there are no guarantees that there will not be complications, but we put (…) chances on our side so that there are as few as possible…” (60-year-old man living with chronic pain and eight other chronic illnesses)

Some illnesses were described by participants as being more severe, more alarming, more dangerous, or more serious than pain. Thus, when uncontrolled, these illnesses could have significant adverse effects on the individual's health and could lead, for example, to hospitalization or life-threatening situations:

“If things become… that can have a negative impact on your health, that can worsen, after that there is pain… of course when that happens, I have to set some priorities, you know. Just shortly before my accident, I take the example of the knee on which the doctors did an X-ray and they told me that the nuts and bolts that were replaced were now in a severe degenerated state. So I have to go back under the knife… Not long after that, I had an accident at the level of my neck, so I put the emphasis on the sequelae of my neck, and all of what that implies, and I put the knee aside a little bit. Especially because it was there that things were the most worrisome.” (40-year-old man living with chronic pain and seven other chronic illnesses)

Thus, the greater health risk associated with these illnesses favored their prioritization by people. However, some other chronic illnesses were considered less alarming or not alarming at all and would therefore be given lower priority in self-management tasks. Pain was often seen as less dangerous or alarming for study participants than some other chronic illnesses, so these characteristics had a greater influence on the prioritization of chronic illnesses other than pain, such as asthma or diabetes, for example.

Also, the level of disability seemed to be another factor considered in the prioritization of chronic illnesses. According to many participants, the most disabling or incapacitating illness was chronic pain, leading to several negative impacts on the person's functioning. Many participants saw their daily lives turned upside down by pain, as it became more complex to carry out activities of daily living, such as walking, cooking, doing household chores, and so forth:

“It has many, many, many impacts… My life, it's always, sit, stand, lie down, sit, stand, lie down… I always have to change position and it's really frustrating…. I cannot stay more than 2 minutes… to walk, it's the same, I cannot… it's even worse, since I fell, I struggle to stand up, my body does not let me, I don't know why…it's intense pain. And my knees, my ankles are going to give up, I'm gonna fall on the ground. The number of times I lost balance, it's crazy, you know, it's constant…” (60-year-old woman living with chronic pain and seven other chronic illnesses)

Indeed, the experience of pain was unpleasant, disturbing, and unbearable, and participants living with chronic pain experienced pain that varied from discomforting to excruciating, leading them to seek relief in order to function: “It was really bothersome to the point where I was telling myself: ‘Will I go to work on Monday?’ Because really, it's bothersome, I sort of have… this incapacity, and it's burdensome.” (52-year-old woman living with chronic pain and two other chronic illnesses) Therefore, to continue to function on a daily basis, chronic pain was often prioritized among other illnesses. Also, most participants described other chronic illnesses as having fewer apparent or bothersome symptoms. Thus, the symptoms of these other chronic illnesses often had less concrete impact on the daily lives of these individuals, since there were fewer symptoms that could disturb daily activities: “Not really symptoms themselves… What stands out is pain.” (57-year-old woman living with chronic pain and two other chronic illnesses). However, some illnesses other than pain did have more apparent and disturbing symptoms, such as the fatigue associated with fibromyalgia, for example: “Pain is bothersome, but feeling that I'm always behind, it's… so that's what bothers me the most, I think, the fatigue… I find that very hard.” (69-year-old woman living with chronic pain and two other chronic illnesses)

*Prioritizing a long-time constant enemy.* Another characteristic that seemed to influence the prioritization of a chronic illness was its omnipresence. Participants frequently expressed that pain was omnipresent, meaning that it was always present in the person's life, that it was always felt by the individual, despite fluctuating intensity. There was no time when pain was not present in these people's daily lives, so pain could take on a greater importance than other chronic illnesses for these participants: “I would say that it takes all of the place… pain… because no matter what you are doing, it's always there!” (40-year-old man living with chronic pain and seven other chronic illnesses) Although other chronic illnesses may remain present in participants’ lives in the long term, these often tend to generate fewer symptoms experienced by participants on a daily basis, as is the case for hypertension or diabetes, for example. Conversely, since pain is almost always experienced by people living with chronic pain, this chronic illness will tend to be given higher priority in the daily management.

*Dealing with social consequences of prioritizing an invisible enemy.* Some study participants mentioned that the “invisibility” of a symptom or chronic illness can have several negative impacts on their daily lives. The invisibility of a symptom or a chronic illness refers to the absence of apparent signs for others, meaning that the symptoms are only perceptible by those who experience them. The two most frequently reported symptoms that were invisible to others by study participants were pain and fatigue. For some participants, the absence of visible pain-related symptoms can make it harder to manage and have social repercussions, as people around them may not understand the reality of living with chronic pain:

“At home, it doesn't really show that I'm in pain, people, you know… I have already learned that… by training, I'm a nurse, I have already learned that… they were saying “ah, people who have pain, it shows… they have facial grimaces”… for me, people cannot say that, so some people are saying “it's not even true that you hurt”, but me, I know however, you know, because you cannot judge people who tell me “you don't hurt”… it's possible, because me, it doesn't show.” (57-year-old woman living with chronic pain and two other chronic illnesses)

Thus, some participants may tend to prioritize pain as it is not visible to others. Indirectly, prioritizing pain may help avoid the social consequences associated with having an invisible chronic illness (e.g., judgment from others, lack of understanding from the social circle, and similar).

3. Psychosocial influences on engagement in and prioritization of self-management

*Social support modulates capacity and motivation to engage in self-management.* Beyond the characteristics of diseases and the stability of prioritization described above, external influences were also demonstrably influencing the extent to which prioritization and engagement in self-management were facilitated or hindered. Several participants emphasized the importance of social support when a person is living with several chronic illnesses. Indeed, a person with a supportive environment will often have easier access to help and support from others, unlike participants with limited social support. This appears to positively influence the capacity and motivation to engage in self-management:

“That, that helped me a lot a lot. I think, in terms of mood, it made a big change because I was in a small city before, I had lost my driver's license because of my health, and I did not have transportation… I was very isolated. Whereas here, at least, I have my sister that I see 2, 3 times per week. They come to pick me up… If I'm missing something, they will help me.” (60-year-old woman living with chronic pain and seven other chronic illnesses)

*Doctor-patient relationship influences uncertainty around self-management.* Equally important for some participants in the self-management of chronic illnesses was the doctor-patient relationship. When this relationship was not meeting the person's needs in terms of resources, knowledge, or understanding, this led to uncertainty in how to optimize self-management: “Before, I had a doctor who was very, very attentive and available. And now, it's no longer the case. So of course, I feel a bit more… left to myself.” (45-year-old woman living with chronic pain and four other chronic illnesses) One factor that can lead to difficulties in the doctor-patient relationship is the invisibility of a symptom, such as pain, as mentioned earlier, since it can make it more difficult for some people to understand the realities associated with chronic pain.

## 3 Discussion

Three main themes were identified from the study narratives: focus on symptom prioritization, key elements of prioritization, and psychosocial influences on engagement in and prioritization of self-management. The first theme, focus on symptom prioritization, showed different prioritization processes, including prioritizing a dominant illness, prioritizing multiple illness to avoid undesirable consequences, and finally absence of or automatic processes of prioritization. In the second theme, we identified several characteristics of an illness, in this case chronic pain that made it a self-management priority: uncontrollable and disabling nature, omnipresence, unpredictability, unpleasantness, and invisibility to others. In the last theme, we highlighted that some psychosocial factors influenced levels of engagement in the self-management and prioritization processes, including social support and the patient-physician relationship.

For some participants, there was a clear focus on a specific aspect of their health, leading them to prioritize a dominant single chronic illness in their daily self-management tasks. This result supports the findings of a narrative literature review conducted by Bratzke et al. (2015), which highlighted that individuals with multiple chronic illnesses consider one chronic illness to be dominant. Moreover, some of the characteristics considered by the authors to determine a dominant disease also aligned with the characteristics detailed in the results of our article to explain illness prioritization, including symptom intensity, disease severity, unpredictable disease course, and impacts on daily life (global outcomes) (Bower et al., 2012; Lindsay, 2009; Schoenberg et al., 2009). We found that pain was the chronic illness most frequently prioritized by participants in their self-management tasks, mostly because it is often difficult to control, unpredictable, unpleasant, and is invisible. Chronic pain was also, for the most part, the chronic illness that had the most negative impact on day-to-day functioning, as it made everyday actions difficult. In the context of multiple concurrent chronic illnesses, these characteristics of chronic pain made this illness stand out for participants in terms of self-management prioritizations, as opposed to more predictable and controllable illnesses such as diabetes.

However, our results have also identified other prioritization profiles; for example the absence of a prioritized illness or the consideration of multiple chronic illnesses as equally dominant. This appeared to be more likely when the prioritized chronic illnesses were both disabling (in terms of functional impacts) and distressing when poorly controlled, underscoring the importance of these two characteristics in establishing priorities among the study participants. Importantly, when participants had equally dominant chronic illnesses, it is often because other illnesses were also perceived as uncontrollable and negatively impacting functioning. These characteristics appear thus central to self-management prioritization decisions.

Social factors were also identified as influencing self-management prioritization process. Beyond its impact on functioning, participants considered its impact on their social relationships, leading them to consider it as a dominant illness to be prioritized. Optimally managed chronic pain could facilitate participants’ engagement in social events and thus help preserve social relationships. This result recalls Deci and Ryan's (2000) self-determination theory, which emphasizes the importance of three fundamental needs in humans: autonomy, competence, and social relationships. Furthermore, it is also important to emphasize that, for some participants, prioritization could be based on social judgment—judgment based on the invisibility of an illness, such as chronic pain, or the fact that it is poorly understood or not recognized by others. Therefore, illnesses were sometimes chosen first in the self-management of various illnesses due to how people perceive them socially.

*Implications of study findings.* The observation that pain is often perceived as dominant compared to other chronic illnesses raises important questions about the self-management strategies to adopt among individuals with multiple chronic illnesses. It is crucial for healthcare professionals helping individuals engage in self-management to consider the presence or not of a dominant illness, such as chronic pain, and adapt strategies accordingly. Many self-management programs for chronic pain have been established, and healthcare providers can support individuals living with chronic pain to adapt to pain chronicity, and manage symptoms, modify roles and responsibilities, and manage emotional challenges associated with pain (Mann et al., 2013). In addition to analgesics, most approaches to pain management include psychological (relaxation, cognitive restructuring, behavioral activation) and physical (exercising, using ice or heat) strategies.

For most study participants, prioritization appeared to be relatively stable, meaning that the same chronic illness was often prioritized from day-to-day. Thus, it might be helpful to initiate self-management education by first understanding the multimorbidity context of an individual and identify ongoing priorities. However, some participants could shift their focus of prioritization in certain situations, especially when a chronic illness became more urgent or alarming for their health. This supports the findings of Morris et al. (2011), who established that the disease considered dominant can fluctuate over time. This also points toward the need to have a self-management plan that is flexible and can adapt to fluctuating prioritization needs. Decisions regarding prioritization in the context of multiple concurrent chronic illnesses should be guided by a comprehensive evaluation of the participants’ health status, considering both functional impact and potential severity of different chronic illnesses.

It is worth noting that chronic illness prioritization, whether it is pain or other chronic illnesses, is often an automatic process rather than a deliberate decision. Healthcare professionals should, therefore, be aware of this automatic prioritization process and provide appropriate information and resources to support individuals in their self-management, making this process more deliberate.

Finally, one participant's comments on the challenges of living with chronic pain highlight the need to address social judgment for people living with invisible chronic illnesses. Raising awareness among healthcare professionals, loved ones, and the community about the realities of individuals living with invisible illnesses, such as chronic pain, may contribute to reducing the social judgment that can ensue.

### 3.1 Limitations

There are some limitations to this study. Study participants were recruited on a voluntary basis for the quantitative component and then selected to represent different numbers of other concurrent chronic illnesses for the qualitative component. As such, the maximum variation sampling procedure did not differentiate participants in terms of types of other chronic illnesses. Participants also responded to a study ad about experiences managing chronic pain in the context of multiple chronic illnesses, which might have influenced types of people interested in participating. We tried to minimize this bias by advertising in many different patient associations and not limited to chronic pain. Furthermore, the sample for the qualitative component predominantly consisted of women, and all participants were Canadians, a limitation to be taken into consideration, since the issues related to chronic pain in the context of multiple concurrent chronic illnesses may potentially differ between men and women and across countries based on healthcare system structure. The participants selected for the qualitative component showed great variability in terms of types of concurrent chronic illnesses. However, the sample did not represent the full range of these possibilities, while prioritization in self-management tasks could possibly vary according to other chronic illnesses. Data collection also took place prior to the COVID-19 pandemic. It is possible that the pandemic altered processes of prioritization and engagement in self-management because of changes to healthcare access and increased limitations on available resources and services. Additionally, the prioritization processes of various chronic illnesses were explored through explicit approaches, such as interviews, while, for several participants, it occurs automatically. In future research on the subject, more implicit methods, such as journaling, could be employed. Lastly, a potential limitation to this study concerns the differentiation between chronic pain and pain associated with other chronic illnesses. During the interviews, participants may have faced challenges in clearly distinguishing these two aspects of pain. This differentiation may not have been adequately emphasized in the interview guide, which could impact how participants understood and shared their pain experiences.

## 4 Conclusions

From this study's findings, we shed light on how and why participants prioritize their chronic illnesses in their daily self-management tasks. The analysis revealed three main themes: focus on symptom prioritization, key elements of prioritization, and psychosocial influences on engagement in and prioritization of self-management.

Pain was a central focus for many participants, but they also acknowledged variations in prioritization strategies based on the characteristics of different chronic illnesses. Understanding these processes can inform healthcare professionals in supporting individuals with chronic illnesses to effectively prioritize their self-management tasks and improve their overall well-being.

## Funding sources

No external funding.

## CRediT authorship contribution statement

**Charlotte Moore-Bouchard:** Writing – original draft, Validation, Methodology, Formal analysis. **Marie-Eve Martel:** Writing – review & editing, Methodology, Data curation. **Elise Develay:** . **José Côté:** Writing – review & editing, Methodology, Conceptualization. **Madeleine Durand:** Writing – review & editing, Validation, Conceptualization. **M Gabrielle Pagé:** .

## Declaration of competing interest

The authors declare that they have no known competing financial interests or personal relationships that could have appeared to influence the work reported in this paper.
